# Evaluation of grain yield, and quality characteristics of some bread wheat cultivars in different agro-ecological regions of Türkiye

**DOI:** 10.1016/j.heliyon.2024.e41547

**Published:** 2024-12-27

**Authors:** Bekir Aktaş, Halil İbrahim Gökdere

**Affiliations:** a Department of Field Crops, Faculty of Agriculture, Yozgat Bozok University, Yozgat, Türkiye; b Variety Registration and Seed Certification Center, Ankara, Türkiye

**Keywords:** *Triticum aestivum* L., Genotype, Environment, *Glu-A1*, *Glu-B1*, *Glu-D1*

## Abstract

Climate change and recurrent droughts challenge wheat production and yield, necessitating careful selection and plant breeding research. "Value for Cultivation and Use" experiments are crucial for assessing genetic gains and providing information about potential pathways to alleviate production losses under specific environmental conditions. The goal of the study was to compare the grain yield and quality characteristics of 46 registered bread wheat cultivars in 5 out of 7 agro-ecological regions of Türkiye between 2016-2017 and 2017–2018. The registered wheat cultivars were also characterized in terms of high molecular weight glutenin subunits (HMW-GS) and the relationships between quality traits and band patterns. Genotype × environment interaction was found statistically significant (*p* < 0.05) for grain yield and the stability of the cultivars as explained on biplot graphs. The Mediterranean and the Central Anatolian region had the highest and the lowest average grain yield of 8137 kg ha^−1^ and 4260 kg ha^−1^ respectively under the rainfed conditions. The average thousand kernel weight of the cultivars was 35.3–39.9 g, with test weight of 77.2–79.2 kg hL^−1^, protein content of 13.4–14.7 %, Zeleny sedimentation of 39.2–53.3 mL, and alveograph energy value varied between 191.2-276.4 × 10^−4^ J. The most common subunits in cultivars were 2∗ at *Glu-A1*, 7 + 8 and 7 + 9 at *Glu-B1*, and 5 + 10 at *Glu-D1*. It is concluded that high-quality new varieties are developed by high-molecular-weight glutenin subunits oriented crosses and selections in Turkish wheat breeding programs.

## 1 Introduction

Wheat is a considerably important annual field crop of economic importance. All wheat breeding programs focus on improving yield and qualitative traits against several stress factors [1]. Changes in meteorological conditions influence wheat yield and quality differently in every agroecological region [2]. Wheat production and yield are challenged by recurrent droughts associated with climate change globally [3]. Therefore, there is need to do selection and plant breeding research based activities very carefully. They underscore the importance of non-genetic environmental factors on the growth, development and yield of would be new variety [4]. Carrying out “Value for Cultivation and Use” experiments have significance importance before registering a new variety [5]. Variations in climatic data influence yield potentials of phenotypes, and genotypes that facilitate them to adapt to regional conditions before they are approved for local cultivation [6,7]. These experiments are performed to assess genetic gains and have significant relevance in breeding experiments. These experiments give possible information and provide information about potential pathways to alleviate losses in production under specific environmental conditions [6]. Wheat production and yield are challenged by recurrent droughts associated with climate change globally.

The yield and quality based performance of all tested genotype in breeding experiments is the result of the interaction among genotypes, and environmental factors [8]. Therefore, evaluating and increasing the yield potential of genotypes in different ecologies in variable climatic conditions is given high importance as an important selection criteria in Turkish wheat breeding programs [9]. Besides these, many other factors like crop rotation, tillage, fertilization, genotype, biotic and abiotic factors could also affect yield and grain yield potential of wheat varieties in a region [10]. Therefore would be new varieties are tested in different environments, to facilitate recommending them for different regions [11] depending on their compatiblity with local ecologies. Some farmers sow wheat during freezing cold in late winter to overcome the challenges of ecology to obtain optimum yield and quality [12,13].

A review of previous literature emphasize significant role of yield components on genetic improvements in bread wheat yield world over. Conversely, these yield gains have tended to require specific and strategic breeding approaches for achieving desired goals of breeding for grain yield and quality [14]. Therefore, understanding and focusing on genotype × environment interactions and their management is of great importance. Kaya and Akcura [15] and Nehe et al. [16] suggest that genotypes and environment including precipitation have the main effect on grain yield and quality characteristics of bread wheat are very important in multilocation experiments [17].

They have significant contribution to understand the behavior of the genotypes during carrying out of breeding experiments to understand their stability and plasticity levels [18]. Furthermore, data from these experimental trials facilitate in easy identification of improved and superior genotypes in final selection for their performance and stability in desirable manner [9].

Gluten and gliadin constitute a large part of the storage proteins in wheat. High molecular weight glutenin subunits had an impact on dough quality in bread making [19], and may be beneficial to plant breeders to improve wheat quality traits [20]. Payne et al. [21] established quality scores according to HMW-GS to predict bread making quality. 5 + 10 encoded by *Glu-D1* had the highest quality scores, 1 and 2∗ encoded by *Glu-A1*, 17 + 18 and 7 + 8 HMW-GS encoded by *Glu-B1* had high quality scores [20,21]. Sonmez et al. [22] suggested that a better understanding of the molecular basis of differences in bread-making quality, genotypes with desired characteristics can be reached faster by screening the plants for the presence of specific alleles (*Glu-A1*, *Glu-B1* and *Glu-D1* loci) for quality-oriented breeding.

Dvoracek et al. [23] reported that electrophoretic evaluation of protein alleles may be suitable for use in the maintenance of cultivars. HMW-GS are used as a supporting element in variety identification and in determining the off-type content of putative cultivars.

Therefore, this study aimed to compare variations in yield, quality characteristics, and HMW-GS level of 46 widely cultivated wheat cultivars during two growing seasons at 34 locations in the Thrace, the Aegean & Southern Marmara, the Mediterranean, the Southeastern Anatolia, and the Central Anatolian conditions (5 agroclimatic regions) for the first time.

## 2 Materials and methods

### 2.1 Experimental locations

**Fig. 1:**
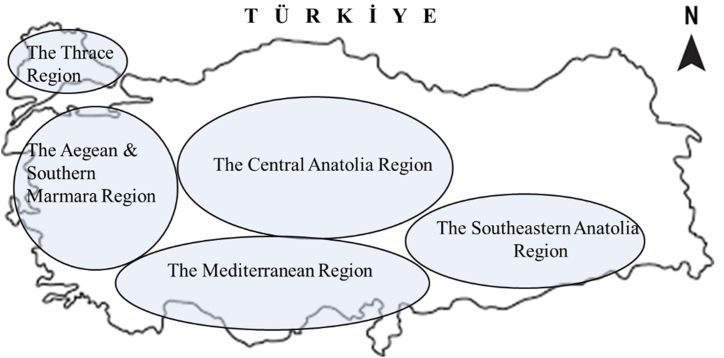
View of the regions on the map of Türkiye where the experiments were conducted.

The trials were carried out at 4 locations in the Thrace region, 7 locations in the Aegean & Southern Marmara region, 5 locations in the Mediterranean region, 7 locations in the Southeastern Anatolia, 5 locations in the Central Anatolian Region under rainfed conditions, and 6 locations in the Central Anatolian Region under the irrigated conditions during 2016-17 and 2017-18. The regions where the experiments were conducted are shown in Fig. 1, and the experiment locations within each region for the first time are mentioned Table 1.

**Table 1:** Experimental locations by region.

The Mediterranean Region	The Aegean&Southern Marmara Region	The Southeastern Anatolia Region	The Central Anatolian Region		The Thrace Region
			Rainfed conditions	Irrigated conditions	
Adana	Adapazari	Adiyaman	Eskisehir	Eskisehir	Edirne
Antalya	Bandirma	Diyarbakir	Gozlu	Gozlu	Kesan
Ceyhan	Dalaman	Kiziltepe	Konya	Karapinar	Luleburgaz
Kahramanmaras	Denizli	Kurtalan	Malya	Konya	Tekirdag
Hatay	Karacabey	Sanliurfa	Ankara	Polatli	
	Menemen	Gundas		Ankara	
	Pamukova	Serince			

### 2.2 Meteorological data

**Fig. 2:**
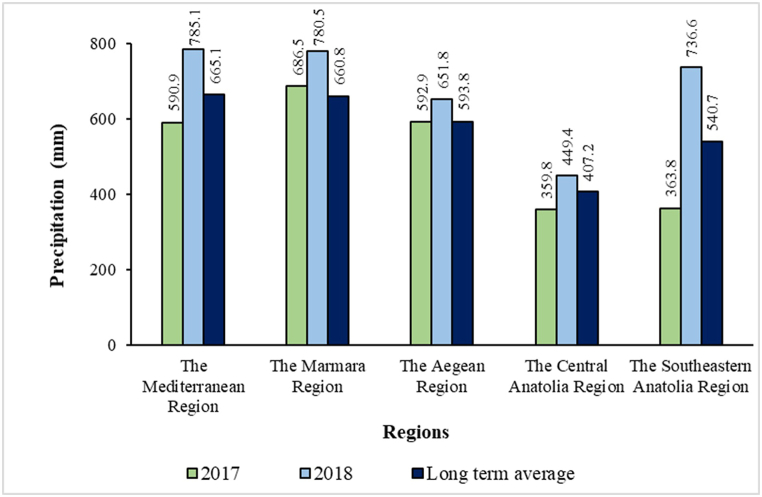
Annual precipitation amounts by region in 2017 and 2018.

Precipitation amounts based on for the years of the study are given [24,25] in Fig. 2. The precipitation was above the long-term averages in all regions except the Marmara region in 2017 and the Southeastern Anatolian region, in general.

### 2.3 Experimental materials

The experimental materials were individually collected from the breeder or variety owner organizations in Türkiye, through the Variety Registration and Seed Certification Centre, Ankara. The putative cultivars used in the experiments are given in Table 2. Winter-type cultivars were evaluated in the Central Anatolia and Thrace regions, and spring-type cultivars were examined in the Aegean & Southern Marmara, The Mediterranean, and Southeastern Anatolia regions. Wheat cultivation in the Central Anatolian region and the Southeastern Anatolian region; were either tested under rainfed or irrigated conditions. In other regions, wheat was grown under rainfed conditions. Since cvs. Ceyhan 99 and Sagittario are widely grown in the Mediterranean, Aegean & Southern Marmara, and Southeastern Anatolia regions, they were used in trials in these three regions. Cv. Esperia was used in The Central Anatolian region and Thrace region trials. Since the cv. Tosunbey was grown in the Central Anatolian region both under rainfed and in irrigated conditions, they were included in both trial sets. Winter season type cv. Pehlivan was evaluated in the Thrace region and also grown in the nearby Southeastern Anatolia region.

**Table 2:** Cultivars used in the study by region.

The Mediterranean Region^a^	The Aegean&Southern Marmara Region^a^	The Southeastern Anatolia Region^b^	The Central Anatolian Region^a^		The Central Anatolian Region^b^	The Thrace Region^a^
Osmaniyem	Hanli	Ceyhan 99	Bezostaja 1		Esperia	Gelibolu
Ceyhan 99	Ziyabey 98	Cemre	Bayraktar 2000		Sultan 95	Pehlivan
Sagittario	Basri Bey 95	Sagittario	Tosunbey		Ahmetaga	Krasunia odes'ka
Pandas	Gonen 98	Pehlivan	Sonmez 2001		Tosunbey	Esperia
Karatopak	Sagittario	Adonis	Demirhan		Konya 2002	Flamura 85
Tekfen 1016	Beskopru	Arin	Ayten Abla		Tekfen 2038	Rumeli
Beyazhan	Ceyhan 99	Polathan	Cavus		Yavuz	Aleppo
	Mirsa				Tugra	Pandiya
					Pecenek	Waximum
					Karakalpak	Anafarta
					Baskurt	Abide
					Bilden	
					Kipcak	

a under the rainfed conditions.

b in the irrigated conditions.

### 2.4 Experimental design and applications

Experiments were conducted in randomized blocks design with 4 replications. Sowing, fertilization, irrigation, weed control, harvesting and quality analyzes were carried out according to the technical protocol for VCU of bread wheat [26]. SDS-PAGE method was used to determine the band patterns of high molecular weight gluten [27]. Quality scores of HMW-GS of cultivars were calculated according to Payne et al. [21].

### 2.5 Statistical analysis

The data were analyzed in the analysis of variance (ANOVA) according to the randomized blocks experimental design with the SAS program [28]. The *F* test was used to determine the statistical significance levels, and an appropriate post hoc test was used to compare the differences among the means. The stability of the cultivars and their vectorial positions were drawn according to the average environment coordinate (AEC). The GGE-biplot method was used and the graphics were drawn with the GenStat package program [29].

## 3 Results

The results of the study for each region were calculated separately and are given in the following lines.

### 3.1 The Mediterranean region

**Fig. 3:**
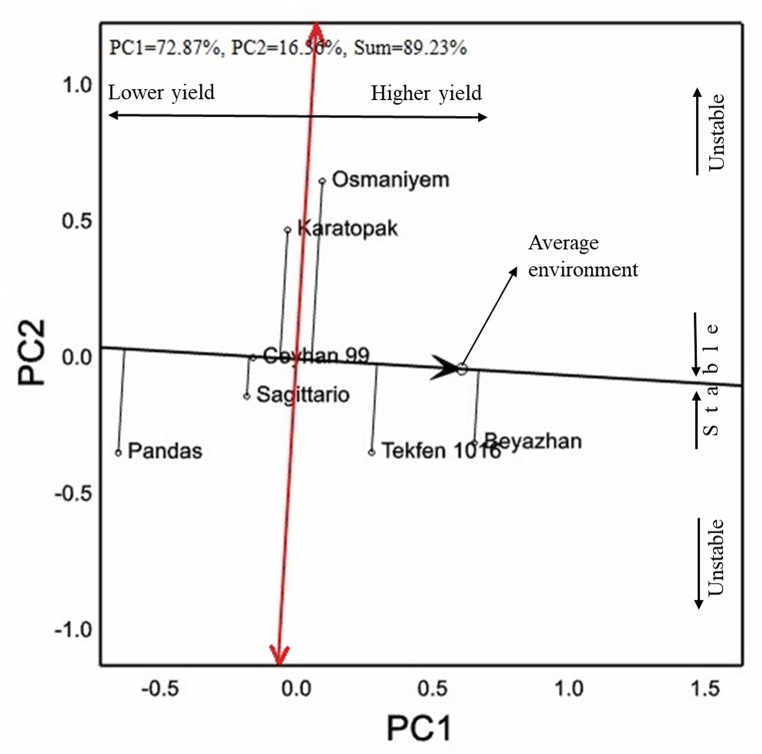
Vector images with respect to AEC of 7 bread wheat cultivars used in the study for grain yield of the Mediterranean region on GGE-biplot graph.

The results from the trials conducted in the Mediterranean Region are shown in Table 3. Seven cultivars were tested in the Mediterranean region. The grain color of 3 cultivars was red and 4 cultivars was white. Cv. Beyazhan had the highest grain yield performance with yield of 9121 kg ha^−1^. Cv. Beyazhan was followed by cv. Tekfen 1016 in yield. Average of all genotypes was determined as 8137 kg ha^−1^. In the GGE-biplot analysis, 89.23 % of the variation was explained and their vectorial appearances according to the average environment coordinate as shown in Fig. 3. Although the position of cv. Ceyhan 99 was above the average environment coordinate apsis, it has a negative PC1 value. Cvs. Beyazhan and Tekfen 1016 had the highest grain yield performance with average environmental coordinate vectors. Cvs. Osmaniyem, Karatopak and Pandas had the longest vectorial position to the average environment coordinate apsis.

**Table 3:** Grain yield, quality parameters, HMW-GS and quality scores of 7 bread wheat cultivars under the rainfed conditions of the Mediterranean region.

Cultivar	GC	GY (kg ha^−1^)	TKW (g)	TW (kg hL^−1^)	PC (%)	ZS (mL)	AEV (10^−4^ J)	*Glu-A1*	*Glu-B1*	*Glu-D1*	*Glu-1* score
Osmaniyem	R	8167^c^	40.6^a^	81.0^a^	14.3^ab^	33.5^d^	224.7	1	7 + 9	5 + 10	6
Ceyhan 99	W	7992^c^	36.2^cd^	78.1^c^	13.2^c^	47.8^bc^	269.0	1	17 + 18	5 + 10	10
Sagittario	R	7982^c^	38.1^bc^	77.6^cd^	14.3^ab^	53.7^ab^	296.3	1	7 + 9	2 + 12	7
Pandas	R	7128^d^	39.2^ab^	76.7^d^	14.4^ab^	50.0^b^	291.7	2∗	7 + 9	5 + 10	9
Karatopak	W	8050^c^	34.3^d^	79.5^b^	14.5^a^	58.7^a^	282.8	1	7 + 8	5 + 10	10
Tekfen 1016	W	8518^b^	35.4^d^	78.8^bc^	14.5^a^	53.7^ab^	297.5	1	17 + 18	2 + 12	8
Beyazhan	W	9121^a^	39.6^ab^	78.8^bc^	14.0^b^	42.5^c^	273.0	2∗	7 + 9	5 + 10	9

Mean		8137	37.6	78.6	14.2	48.6	276.4				
*F*		∗∗	∗∗	∗∗	∗∗	∗∗	ns				
*CV* (%)		8.4	5.1	1.4	2.6	10.8	17.8				

∗∗, significant at *p* < 0.01; ns, non-significant; R, red; W, white; GC, grain color; GY, grain yield; TKW, thousand kernel weight; TW, test weight; PC, protein content; ZS, Zeleny sedimentation; AEV, alveograph energy value.

Cv. Osmaniyem is the most prominent cultivar with the highest thousand kernel weight and test weight. Except for the cv. Ceyhan 99, the other cultivars had the protein content values in the range of 14.0–14.5 %. Cvs. Karatopak, Tekfen 1016 and Sagittario showed the highest values for Zeleny sedimentation. There was no statistical difference among cultivars in alveograph energy value. Subunit 1 in five cultivars and subunit 2∗ in two cultivars were determined at the *Glu-A1* locus in terms of HMW-GS. Predominantly genotypes had subunit 7 + 9 at *Glu-B1* and subunit 5 + 10 at *Glu-D1*. These genotypes had values ranging between 6 and 10 in terms of quality scores (given according to HMW-GS). Since cv. Osmaniyem has the 1B/1R rye translocation, 3 points were removed from the total quality score calculation [16]. Cv. Osmaniyem has a value of 33.5 mL in Zeleny sedimentation, well below the other cultivars.

### 3.2 The Aegean & Southern Marmara region

**Fig. 4:**
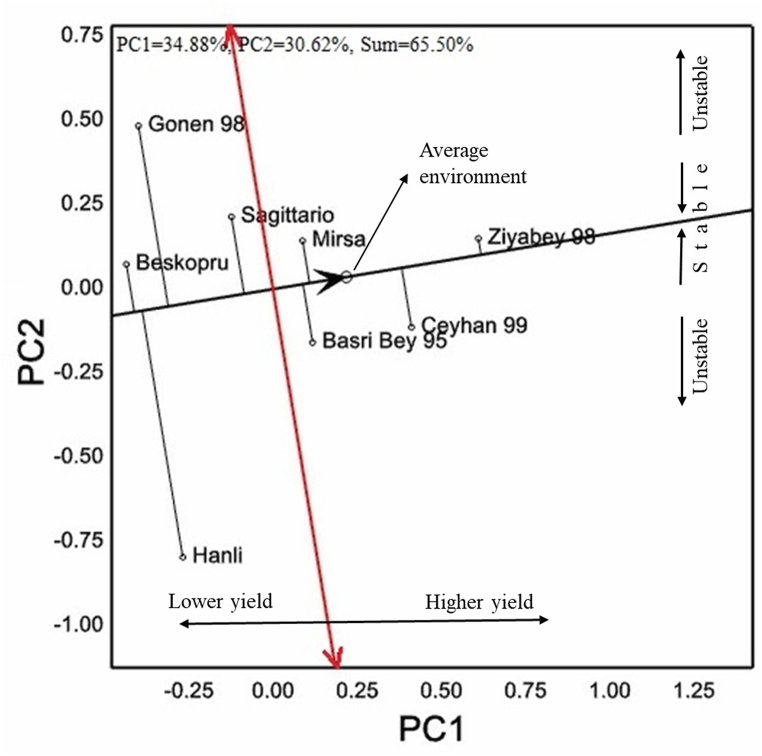
Vector images with respect to AEC of 8 bread wheat cultivars used in the study for grain yield of the Aegean & Southern Marmara region on GGE-biplot graph.

Eight cultivars were evaluated in this region. Five cultivars had white and 3 had red grain color, and cv. Ziyabey 98 ranked first with a grain yield of 7192 kg ha^−1^ (Table 4) in the Aegean & Southern Marmara Region trials. 65.50 % of the variation was explained by GGE-biplot analysis (Fig. 4). Cv. Ziyabey 98 had the highest grain yield performance. The performance was stable depending on its location close to the average environmental coordinate apsis. Especially cvs. Hanli and Gonen 98 had very long vectorial lengths with average environmental coordinate, which showed their low stability.

**Table 4:** Grain yield, quality parameters, HMW-GS and quality scores of 8 bread wheat cultivars under the rainfed conditions of the Aegean & Southern Marmara region.

Cultivar	GC	GY (kg ha^−1^)	TKW (g)	TW (kg hL^−1^)	PC (%)	ZS (mL)	AEV (10^−4^ J)	*Glu-A1*	*Glu-B1*	*Glu-D1*	*Glu-1* score
Hanli	R	6411^de^	37.2^bc^	78.9^bc^	13.4	44.2^bc^	206.6^ab^	2∗	7 + 8	5 + 10	10
Ziyabey 98	W	7192^a^	38.6^abc^	77.3^d^	13.2	40.0^c^	120.8^c^	2∗	7	5 + 10	8
Basri Bey 95	W	6683^bc^	34.2^d^	78.2^cd^	13.2	30.6^d^	162.8^bc^	2∗	7 + 9	5 + 10	9
Gonen 98	W	6335^e^	36.7^cd^	78.3^bcd^	13.5	46.0^bc^	185.2^ab^	2∗	17 + 18	2 + 12	8
Sagittario	R	6652^bcd^	40.1^ab^	77.2^d^	13.9	58.2^a^	225.0^a^	1	7 + 9	2 + 12	7
Beskopru	R	6453^cde^	39.9^ab^	79.9^b^	13.6	54.8^ab^	185.0^ab^	1	7 + 9	5 + 10	9
Ceyhan 99	W	6753^b^	39.8^ab^	79.0^bc^	13.0	46.2^bc^	205.0^ab^	1	17 + 18	5 + 10	10
Mirsa	W	6801^b^	41.2^a^	81.8^a^	13.5	47.0^bc^	239.4^a^	1	17 + 18	5 + 10	10

Mean		6660	38.5	78.8	13.4	45.9	191.2				
*F*		∗∗	∗∗	∗∗	ns	∗∗	∗				
*CV* (%)		9.7	5.7	1.6	4.6	18.2	24.9				

∗, Significant at *p* < 0.05; ∗∗, significant at *p* < 0.01; ns, non-significant; R, red; W, white; GC, grain color; GY, grain yield; TKW, thousand kernel weight; TW, test weight; PC, protein content; ZS, Zeleny sedimentation; AEV, alveograph energy value.

Cv. Mirsa with a 1000 kernel weight and test weight with the highest values. All cultivars were collected in 2 statistical groups, which showed a narrow range of distribution in protein contents. Although cvs. Sagittario and Beskopru had the highest values above 50 mL in Zeleny sedimentation. Cv. Basri Bey 95 showed the lowest sedimentation value among the cultivars. Cv. Mirsa, had the highest values in terms of physical quality characteristics (thousand kernel weight and test weight), which ranked first in alveograph energy value, and cv. Ziyabey 98, which showed the highest value in grain yield, and the lowest alveograph energy value. Subunit 1 in four genotypes and subunit 2∗ in the other four genotypes were determined at *Glu-A1* locus, and the majority of genotypes; which had subunit 5 + 10 at *Glu-D1* locus. At the *Glu-B1* locus, cultivars exhibited 7, 7 + 8, 7 + 9 and 17 + 18 band patterns. All genotypes had values in between 7 and 10 using *Glu-1* quality scores.

### 3.3 The Southeastern Anatolia Region

**Fig. 5:**
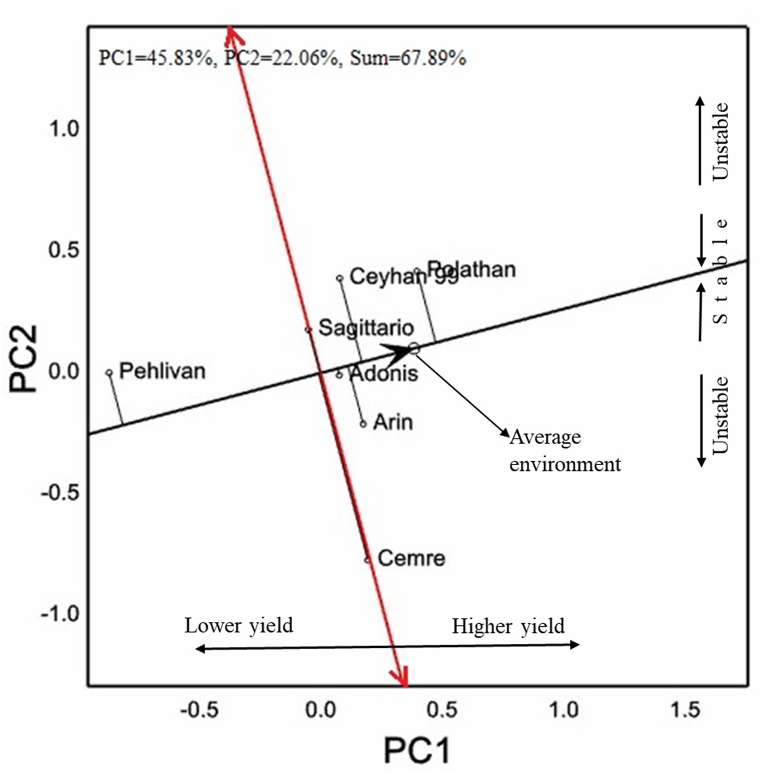
Vector images with respect to AEC of 7 bread wheat cultivars used in the study for grain yield of the Southeastern Anatolia region on GGE-biplot graph.

Seven genotypes were studied in this region. Four genotypes had white and three genotypes had red grain color in the experiments carried out under irrigated conditions of the Southeastern Anatolia Region (Table 5). Cv. Polathan is in the first place with 7026 kg ha^−1^ and cv. Pehlivan is in the last place with 5693 kg ha^−1^ in grain yield. Cv. Pehlivan was well below the other genotypes with grain yield value. In the GGE-biplot analysis, 67.89 % of the variation was observed, of which 45.83 % was distributed in PC1 and 22.06 % in PC2 (Fig. 5). The cultivar with the highest PC1 score was cvs. Polathan, and Adonis located very close to the average environmental coordinate apsis.

**Table 5:** Grain yield, quality parameters, HMW-GS and quality scores of 7 bread wheat cultivars in the irrigated conditions of the Southeastern Anatolia region.

Cultivar	GC	GY (kg ha^−1^)	TKW (g)	TW (kg hL^−1^)	PC (%)	ZS (mL)	AEV (10^−4^ J)	*Glu* *A1*	*Glu* *B1*	*Glu* *D1*	*Glu-1* score
Ceyhan 99	W	6725^b^	37.3^b^	79.2^b^	13.9^c^	43.2	320.8^a^	1	17 + 18	5 + 10	10
Cemre	W	6633^b^	42.8^a^	78.6^c^	14.9^b^	38.0	262.4^bc^	2∗	17 + 18	2 + 12	8
Sagittario	R	6694^b^	39.5^ab^	78.7^b^	15.0^b^	38.2	287.0^ab^	1	7 + 9	2 + 12	7
Pehlivan	R	5693^c^	42.8^a^	79.5^b^	13.8^c^	35.2	226.0^c^	2∗	7 + 9	2 + 12	7
Adonis	W	6649^b^	39.7^ab^	78.7^b^	16.0^a^	41.0	245.6^bc^	2∗	7 + 9	5 + 10	9
Arin	R	6446^b^	35.1^c^	80.8^a^	14.9^b^	37.0	214.2^c^	2∗	7 + 9	2 + 12	7
Polathan	W	7026^a^	42.3^a^	79.0^b^	14.6^bc^	41.6	270.6^abc^	1	7 + 9	5 + 10	9

Mean		6552	39.9	79.2	14.7	39.2	260.9				
*F*		∗∗	∗∗	∗∗	∗∗	ns	∗				
*CV* (%)		10.9	6.6	1.0	4.8	15.0	16.9				

∗, Significant at *p* < 0.05; ∗∗, significant at *p* < 0.01; ns, non-significant; R, red; W, white; GC, grain color; GY, grain yield; TKW, thousand kernel weight; TW, test weight; PC, protein content; ZS, Zeleny sedimentation; AEV, alveograph energy value.

Cvs. Cemre, Pehlivan, and Polathan had the highest values at a 1000 kernel weight. While cv. Arin had the lowest value (35.1 g) in 1000 kernel weight, it was the genotype with the highest value (80.8 kg hL^−1^) in test weight. Protein content of cv. Adonis was well above other genotypes with a value of 16.0 %. Cvs. Ceyhan 99 and Pehlivan were the two genotypes with protein contents below 14.0 %. The average of the cultivars was 39.2 mL, and no statistical significance was found among the genotypes in terms of Zeleny sedimentation. Cv. Ceyhan 99, showed a value of 320.8 × 10^−4^ J which also showed the maximum alveograph energy value performance in The Mediterranean and Aegean & Southern Marmara Regions. The cultivars had a *Glu-1* quality score between 7 and 10 according to the band patterns.

### 3.4 The Central Anatolian region

**Fig. 6:**
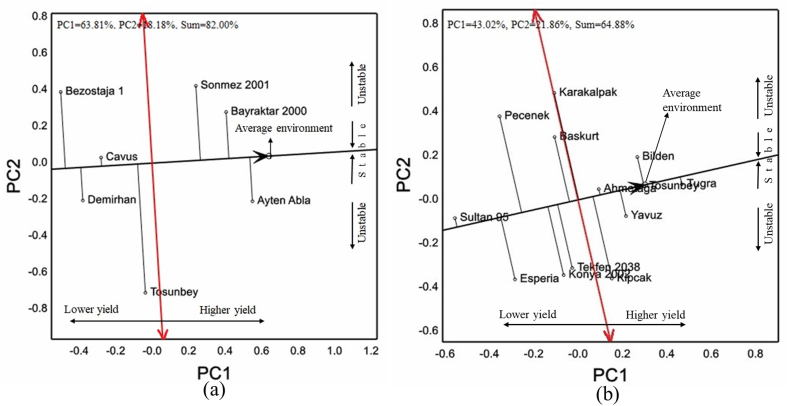
Vector images with respect to AEC of bread wheat cultivars used in the study for grain yield (a) under the rainfed and (b) irrigated conditions of the Central Anatolian Region with GGE-biplot graph.

The average grain yield of the genotypes was 4260 kg ha^−1^ in the trials in which 7 cultivars were included under the rainfed conditions of the Central Anatolian Region (Table 6). Cvs. Ayten Abla and Bayraktar 2000 showed the highest grain yield. The analysis of variance, GGE-biplot analysis explained 82.0 % of variation, of which 63.81 % was distributed in PC1 and 18.18 % in PC2 (Fig. 6a). In general, the genotypes had long vector lengths with average environmental coordinate. Although the vector lengths of the cvs. Ayten Abla and Bayraktar 2000 showed high performance in grain yield, they were similar. The cv. Ayten Abla had a negative PC2 value.

**Table 6:** Grain yield, quality parameters, HMW-GS and quality scores of 7 bread wheat cultivars under the rainfed conditions of the Central Anatolian Region.

Cultivar	GC	GY (kg ha^−1^)	TKW (g)	TW (kg hL^−1^)	PC (%)	ZS (mL)	AEV (10^−4^ J)	*Glu* *A1*	*Glu* *B1*	*Glu* *D1*	*Glu-1* score
Bezostaja 1	R	3654^e^	39.2^a^	77.3	15.3^a^	46.5^b^	257.8^bc^	2∗	7 + 9	5 + 10	9
Bayraktar 2000	W	4768^ab^	38.3^a^	77.2	13.6^b^	34.5^cd^	161.0^d^	2∗	7 + 8	5 + 10	10
Tosunbey	W	4155^c^	33.7^b^	77.0	14.6^ab^	42.3^bc^	297.3^b^	1	17 + 18	5 + 10	10
Sonmez 2001	R	4610^b^	39.9^a^	77.5	13.6^b^	32.5^d^	188.8^cd^	1	7	2 + 12	6
Demirhan	R	3761^d^	33.5^b^	77.6	15.2^a^	46.3^bc^	253.0^bc^	1	7 + 8	5 + 10	10
Ayten Abla	R	4951^a^	32.6^b^	77.9	14.1^ab^	40.8^bcd^	219.0^bcd^	null	7 + 9	2 + 12	5
Cavus	R	3924^cd^	34.6^b^	77.8	15.4^a^	59.8^a^	433.0^a^	2∗	7 + 9	5 + 10	9

Mean		4260	36.0	77.5	14.5	43.2	258.6				
*F*		∗∗	∗∗	ns	∗	∗∗	∗∗				
*CV* (%)		16.1	5.4	1.0	6.6	18.5	20.7				

∗, Significant at *p* < 0.05; ∗∗, significant at *p* < 0.01; ns, non-significant; R, red; W, white; GC, grain color; GY, grain yield; TKW, thousand kernel weight; TW, test weight; PC, protein content; ZS, Zeleny sedimentation; AEV, alveograph energy value.

Cvs. Sonmez 2001, Bezostaja 1, and Bayraktar 2000 showed high values in thousand kernel weight and were in the same statistical group. Statistical significance was not determined among genotypes in test weight. In terms of protein contents, the genotypes were distributed in two separate statistical groups. Cvs. Cavus, Bezostaja 1, and Demirhan showed protein contents of over 15 %. Large differences were determined among genotypes in Zeleny sedimentation and alveograph energy value. Cv. Cavus showed very high average values in terms of both parameters. Cvs. Sonmez 2001 and Bayraktar 2000 had the lowest values between 5 and 10 in terms of *Glu-1* quality scores. Cv. Ayten Abla had 5 *Glu-1* quality scores with n, 7 + 9, and 2 + 12 band patterns.

In the experiments carried out with 13 genotypes under irrigated conditions in the Central Anatolian Region, two genotypes were white and the others had red grain color (Table 7). The genotypes showed grain yield averages between 6355 and 7485 kg ha^−1^. GGE-biplot analysis explained 64.9 % variation (Fig. 6b). Cvs. Tugra, Tosunbey, and Ahmetaga had positions close to the apsis of the average environmental coordinate or their short vector lengths; which indicated high stability. Although cvs. Bilden and Yavuz had relatively longer vectors, they had positive PC1 scores.

**Table 7:** Grain yield, quality parameters, HMW-GS and quality scores of 13 bread wheat cultivars under irrigated conditions of the Central Anatolian region.

Cultivar	GC	GY (kg ha^−1^)	TKW (g)	TW (kg hL^−1^)	PC (%)	ZS (mL)	AEV (10^−4^ J)	*Glu* *A1*	*Glu* *B1*	*Glu* *D1*	*Glu-1* score
Esperia	R	6494^fg^	34.3^d-g^	76.8^bcd^	14.5	53.0^ab^	279.0^a^	2∗	7 + 8	5 + 10	10
Sultan 95	W	6355^g^	30.1^h^	73.7^e^	14.3	41.2^cde^	154.2^de^	2∗	7	5 + 10	8
Ahmetaga	R	7243^abc^	31.6^fgh^	77.1^a-d^	14.1	54.8^a^	228.2^abc^	1	7 + 8	5 + 10	10
Tosunbey	W	7299^ab^	34.2^d-g^	78.1^abc^	14.2	42.6^cde^	232.0^abc^	1	17 + 18	5 + 10	10
Konya 2002	R	6786^d-g^	41.6^a^	79.2^a^	13.5	39.4^cde^	189.4^cde^	2∗	7 + 9	2 + 12	7
Tekfen 2038	R	6839^c-f^	34.9^c-f^	77.4^abc^	13.7	53.2^ab^	275.4^ab^	2∗	7 + 8	5 + 10	10
Yavuz	R	7188^a-d^	36.5^cde^	77.6^abc^	13.2	36.6^def^	208.4^bcd^	2∗	7 + 8	5 + 10	10
Tugra	R	7485^a^	34.9^c-f^	78.2^ab^	13.7	38.0^c-f^	202.8^cde^	1	7	5 + 10	8
Pecenek	R	6675^efg^	31.2^gh^	75.8^cde^	14.1	55.4^a^	218.2^a-d^	null	17 + 18	2 + 12	6
Karakalpak	R	6802^def^	37.3^bcd^	74.8^de^	13.7	30.8^f^	168.4^cde^	1	7 + 9	2 + 12	7
Baskurt	R	7020^b-e^	40.3^ab^	77.8^abc^	13.7	45.8^bc^	191.8^cde^	1	7 + 8	5 + 10	10
Bilden	R	7427^ab^	38.2^bc^	78.7^ab^	13.8	36.2^ef^	135.8^e^	2∗	7 + 9	5 + 10	9
Kipcak	R	7049^a-e^	33.3^e-h^	78.6^ab^	13.0	44.4^cd^	211.6^a-d^	2∗	7 + 8	5 + 10	10

Mean		6974	35.3	77.2	13.8	44.0	207.3				
*F*		∗∗	∗∗	∗∗	ns	∗∗	∗∗				
*CV* (%)		12.8	7.6	2.3	6.7	14.1	26.0				

∗∗, significant at *p* < 0.01; ns, non-significant; R, red; W, white; GC, grain color; GY, grain yield; TKW, thousand kernel weight; TW, test weight; PC, protein content; ZS, Zeleny sedimentation; AEV, alveograph energy value.

While cv. Konya 2002 had the highest values in 1000 kernel weight and test weight, cv. Sultan 95 showed the lowest average values. No statistically significant difference was found among genotypes in protein contents. Cvs Pecenek, Ahmetaga, Tekfen 2038, and Esperia had Zeleny sedimentation values above 50 mL. Cvs. Esperia and Tekfen 2038 had the highest values in alveograph energy value, while cv. Bilden had the lowest value of 135.8 × 10-4 J. All genotypes had *Glu-1* quality scores between 6 and 10. Cvs. Esperia, Ahmetaga, Tosunbey, Tekfen 2038, Yavuz, Baskurt, and Kipcak had quality score of 10. The *Glu-A1* locus of cv. Pecenek had no band and scored 6.

### 3.5 The Thrace Region

**Fig. 7:**
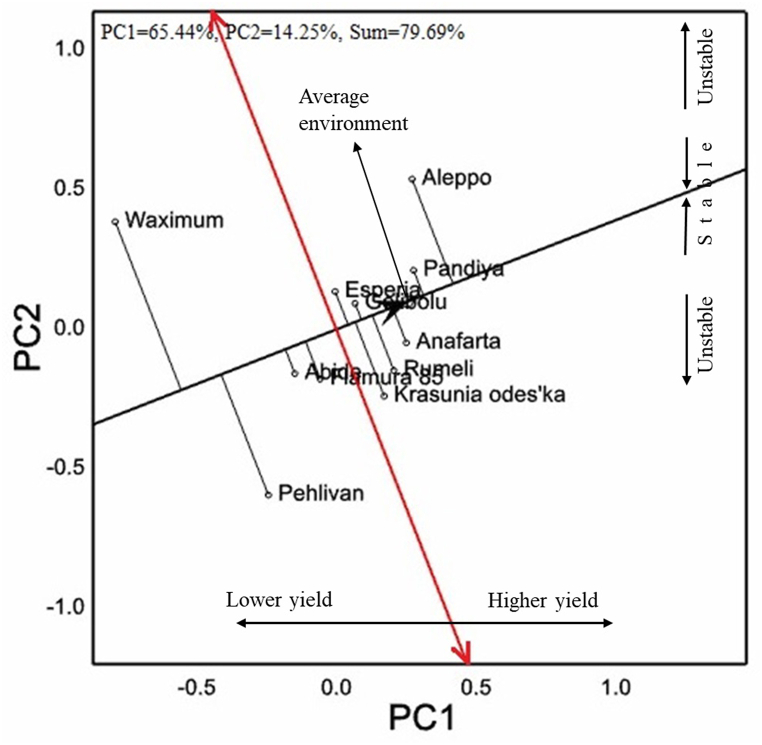
Vector images with respect to AEC of 11 bread wheat cultivars used in the study for grain yield of wheat in the Thrace region on GGE-biplot graph.

Eleven cultivars were tested in the Thrace Region. Cv. Aleppo had white grain color and all other cultivars had red grain color (Table 8). Cvs. Aleppo (with a yield of 7544 kg ha^−1^) and Pandiya (with a yield of 7308 kg ha^−1^) were the best-performing cultivars for their grain yield. Cv. Waximum, a waxy cultivar, had the lowest grain yield of 6167 kg ha^−1^. By GGE-biplot analysis, 79.69 % of the variation was explained, of which 65.44 % is represented in PC1 (Fig. 7). While cvs. Pehlivan and Waximum had low grain yield performances, they did not show a stable appearance that moved away from the average environmental coordinate apsis maximum. Although cv. Aleppo had the highest grain yield performance, its binding to average environmental coordinate with a long vector indicates a negative situation in terms of stability. Cv. Pandiya, with the highest performance after cv. Aleppo, showed it was a stable cultivar with its close location to average environmental coordinate.

**Table 8:** Grain yield, quality parameters, HMW-GS and quality scores of 11 bread wheat cultivars under the rainfed conditions of the Thrace region.

Cultivar	GC	GY (kg ha^−1^)	TKW (g)	TW (kg hL^−1^)	PC (%)	ZS (mL)	AEV (10^−4^ J)	*Glu* *A1*	*Glu* *B1*	*Glu* *D1*	*Glu-1* score
Gelibolu	R	7069^bcd^	38.2^bc^	78.5^a^	13.6^ef^	49.4^de^	189.3^de^	2∗	7 + 8	2 + 12	8
Pehlivan	R	6390^f^	42.9^a^	78.4^ab^	14.3^b-e^	40.8^f^	188.0^de^	2∗	7 + 9	2 + 12	7
Krasunia odes'ka	R	6930^cde^	36.9^bcd^	77.3^abc^	13.2^f^	57.0^bc^	236.6^bcd^	2∗	7 + 8	5 + 10	10
Esperia	R	6916^cde^	32.7^e^	76.2^cd^	15.0^ab^	62.8^ab^	270.2^ab^	2∗	7 + 8	5 + 10	10
Flamura 85	R	6788^e^	39.0^b^	79.2^a^	14.6^bc^	52.2^cd^	212.2^cde^	2∗	7 + 8	5 + 10	10
Rumeli	R	7147^bc^	35.5^cde^	79.3^a^	15.7^a^	66.6^a^	307.0^a^	2∗	7 + 8	5 + 10	10
Aleppo	W	7544^a^	26.3^f^	77.8^abc^	14.5^bcd^	53.0^cd^	258.8^abc^	null	7 + 8	2 + 12	6
Pandiya	R	7308^ab^	35.3^de^	76.4^bcd^	14.3^b-e^	58.8^bc^	206.6^cde^	2∗	7 + 8	5 + 10	10
Waximum	R	6167^f^	29.0^f^	72.2^e^	13.9^c-f^	57.4^bc^	240.8^bcd^	1	17 + 18	2 + 12	8
Anafarta	R	7135^bc^	36.9^bcd^	75.3^d^	13.8^def^	44.4^ef^	184.8^de^	null	6 + 8	5 + 10	6
Abide	R	6862^de^	36.8^bcd^	78.5^a^	13.7^def^	43.6^ef^	176.0^e^	2∗	7 + 8	5 + 10	10

Mean		6932	35.4	77.2	14.2	53.3	224.6				
*F*		∗∗	∗∗	∗∗	∗∗	∗∗	∗∗				
*CV* (%)		7.4	6.3	2.1	4.4	10.1	20.0				

∗∗, significant at *p* < 0.01; ns, non-significant; R, red; W, white; GC, grain color; GY, grain yield; TKW, thousand kernel weight; TW, test weight; PC, protein content; ZS, Zeleny sedimentation; AEV, alveograph energy value.

Cv. Pehlivan had the highest value in terms of thousand kernel weight, cvs. Aleppo and Waximum had the lowest values under 30 g. While cvs. Rumeli and Flamura 85 showed average values over 79 kg hL^−1^ in test weight, cv. Waximum showed the lowest value of 72.2 kg hL^−1^. Cv. Rumeli had the highest performance with 15.7 % protein content, followed by cv. Esperia with 15.0 % value. Cv. Rumeli had the highest values in test weight and protein contents. These differed from other cultivars in terms of Zeleny sedimentation and alveograph energy values that showed very high values. *Glu-1* quality scores, and genotypes had values between 6 and 10. Six genotypes had a score of 10 and two had a score of 6.

### 3.6 Grain yield and quality parameters on the basis of regions

Averages and standard deviations of grain yield and quality parameters are given in Table 9. The Mediterranean Region ranked first in grain yield with 8137 kg ha^−1^. The Central Anatolian Region (under the rainfed conditions) showed the lowest grain yield performance with 4260 kg ha^−1^. The highest standard deviation values were determined in The Mediterranean and The Central Anatolian dry conditions. Other regions showed average grain yield values between 6552 and 6974 kg ha^−1^. Southeastern Anatolia Region had the highest average values in thousand kernel weight and test weight. The Aegean & Southern Marmara and The Central Anatolian Region (irrigated conditions) had an average protein content less than 14 %, while other regions showed values above this. The overall average in Zeleny sedimentation was 45.7 mL, and Thrace, The Mediterranean and the Aegean & Southern Marmara Regions had values above the average. The Mediterranean Region showed the highest average in alveograph energy value, followed by Southeastern Anatolia (irrigated conditions) and The Central Anatolian Regions (rainfed conditions). The Aegean & Southern Marmara Region were the only regions with an alveograph energy value below 200 × 10^−4^ J.

**Table 9:** Grain yield and quality characteristics' average values and standard deviations by regions.

Region	GY (kg ha^−1^)	TKW (g)	TW (kg hL^−1^)	PC (%)	ZS (mL)	AEV (10^−4^ J)
The Mediterranean^a^	8137 (604)^c^	37.6 (2.4)	78.6 (1.4)	14.2 (0.5)	48.6 (8.4)	276.4 (25.3)
Aegean & Southern Marmara^a^	6660 (273)	38.5 (2.3)	78.8 (1.5)	13.4 (0.3)	45.9 (8.5)	191.2 (37.3)
Southeastern Anatolia^b^	6552 (416)	39.9 (3.0)	79.2 (0.8)	14.7 (0.7)	39.2 (2.8)	260.9 (36.5)
Central Anatolia^a^	4260 (516)	36.0 (3.0)	77.5 (0.3)	14.5 (0.8)	43.2 (9.1)	258.6 (89.4)
Central Anatolia^b^	6974 (352)	35.3 (3.4)	77.2 (1.6)	13.8 (0.4)	44.0 (8.0)	207.3 (41.8)
Thrace^a^	6932 (391)	35.4 (4.6)	77.2 (2.1)	14.2 (0.7)	53.3 (8.2)	224.6 (41.8)

Mean	6586	37.1	78.1	14.1	45.7	236.5

a under the rainfed conditions.

b under the irrigated conditions.

c data in brackets are standard deviation; GY, grain yield; TKW, thousand kernel weight; TW, test weight; PC, protein content; ZS, Zeleny sedimentation; AEV, alveograph energy value.

### 3.7 Grain yield and quality parameters in terms of Glu-1 scores

**Fig. 8:**
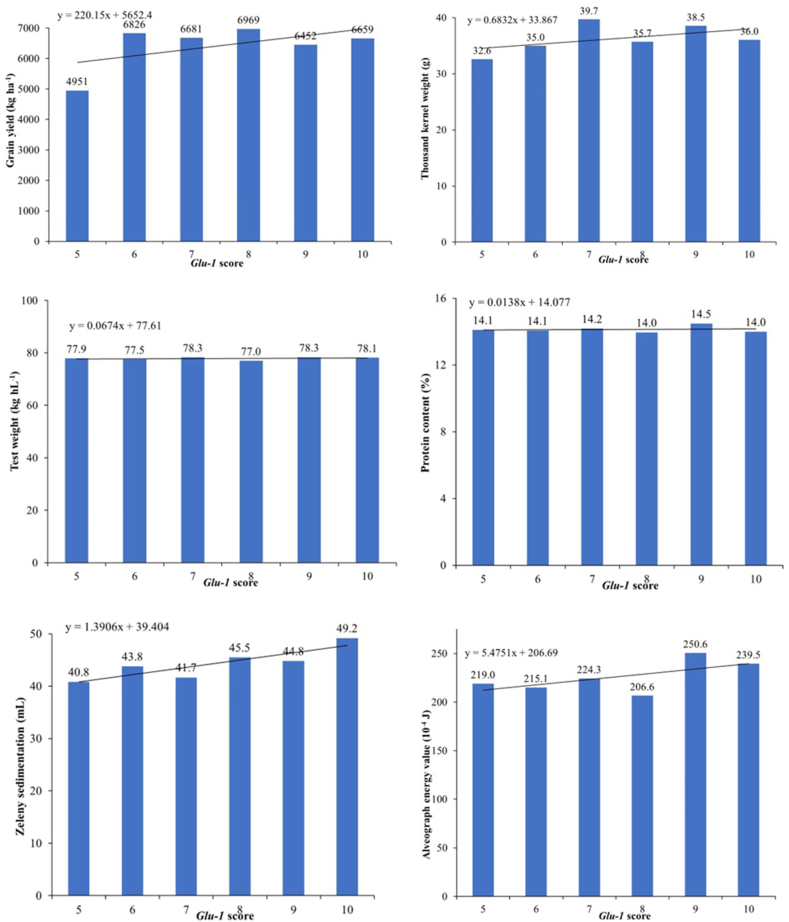
The average values of the attributes assessed based on the *Glu-1* quality scores.

The average values of the traits examined according to the *Glu-1* quality scores calculated on the basis of the HMW-GS are given in Table 10. 18 cultivars had a *Glu-1* quality score of 10, while only one cultivar had a *Glu-1* quality score of 5 among 46 genotypes used in this study. When the test weight and protein content graphs (Fig. 8), it was observed that that the slope line was almost horizontal and did not show an increasing tendency according to *Glu-1* scores. It was observed that there was an increasing trend in other parameters.

**Table 10:** Average values of grain yield and quality traits of *Glu-1* scores.

*Glu-1* score	F	GY (kg ha^−1^)	TKW (g)	TW (kg hL^−1^)	PC (%)	ZS (mL)	AEV (10^−4^ J)
5	1	4951	32.6	77.9	14.1	40.8	219.0
6	5	6826	35.0	77.5	14.1	43.8	215.1
7	5	6681	39.7	78.3	14.2	41.7	224.3
8	8	6969	35.7	77.0	14.0	45.5	206.6
9	9	6452	38.5	78.3	14.5	44.8	250.6
10	18	6659	36.0	78.1	14.0	49.2	239.5

F, Frequency; GY, grain yield; TKW, thousand kernel weight; TW, test weight; PC, protein content; ZS, Zeleny sedimentation; AEV, alveograph energy value.

The loci and allele numbers observed in 46 cultivars according to HMW-GS are shown in Table 11. While the highest subunit 2∗ was observed at the *Glu-A1* locus of 26 cultivars, subunit 1 was determined in 16 genotypes. Null allele was observed at the *Glu-A1* locus of 4 genotypes. The highest alleles at the *Glu-B1* locus were observed for subunits 7 + 8 and 7 + 9. Subunit 17 + 18 was determined in 8 genotypes only; subunit 7 was noted in 4 genotypes and subunit 6 + 8 was noted in one genotype only. 32 genotypes carried subunit 5 + 10 at the *Glu-D1* locus, subunit 2 + 12 was observed in 14 genotypes. The HMW-GS did not show a large variation in protein content averages. In Zeleny sedimentation, the highest average was noted in 7 + 8 subunits. The alveograph energy value showed the highest average of 17 + 18 subunits. 7 and 6 + 8 are subunits that showed an alveograph energy value below 200 × 10^−4^ J.

**Table 11:** Allele numbers and the effect of HMW-GS on grain yield and quality parameters studied on bread wheat cultivars.

Glu locus	Subunit	Allele numbers	GY (kg ha^−1^)	TKW (g)	TW (kg hL^−1^)	PC (%)	ZS (mL)	AEV (10^−4^ J)
*Glu-A1*	null	4	6576.3	31.8	76.7	14.1	48.4	220.2
	1	16	6778.8	37.1	78.1	14.0	46.1	243.2
	2∗	26	6578.0	37.3	78.0	14.1	45.7	224.3
*Glu-B1*	7	4	6410.5	35.9	76.7	13.7	37.9	166.7
	17 + 18	8	6732.1	36.1	77.9	13.9	47.2	251.6
	6 + 8	1	7135.0	36.9	75.3	13.8	44.4	184.8
	7 + 8	17	6743.7	35.3	77.9	14.1	50.8	230.8
	7 + 9	16	6559.4	38.8	78.4	14.3	42.7	236.5
*Glu-D1*	2 + 12	14	6585.1	37.0	77.7	14.1	44.7	226.7
	5 + 10	32	6691.6	36.7	78.0	14.1	46.7	233.7

GY, grain yield; TKW, thousand kernel weight; TW, test weight; PC, protein content; ZS, Zeleny sedimentation; AEV, alveograph energy value.

## 4 Discussion

### 4.1 Grain yield

Environmental variation in yield and quality traits is more prominent than genotypic variation [15,30]. The variation in grain yield was mainly caused by the environment which varied according to the areas, with the share of genotype and genotype × environment interaction around 20 %. Precipitation is a more determining factor in the yield of bread wheat compared to other factors [31,32]. It can be said that the precipitation amounts of each region in Fig. 2 and the grain yields in Table 9 increased proportionally. A significant difference was found among all genotypes in their average grain yield under irrigated and dry conditions. The Central Anatolian region has the lowest grain yield under the rainfed conditions if all regions are compared. The limits of genetic performance are being challenged, showing the need for an increase in irrigable areas with the need to increase genotype resistance against biotic and abiotic stresses [33].

In addition to environmental factors, the effects of the genotypes used in the study and the interaction of these genotypes with the environment should not be ignored. Therefore, GGE-biplot graphs for the grain yield of the genotypes were also created. Türkiye's the agro-geographical regions mentioned in this study had significantly distinguished ecologies showing inter and intra effects, on wheat cultivars. Climate change has caused disturbances in heat, drought, and moisture levels [[34], [35], [36], [37]]. This has resulted in excabarated spring frost damage to winter wheat with negative effects on crop development [38]. Long vectors of some genotypes to average environmental coordinate showed that variable genotype × environment interaction was an influential factor within each region. Yan and Kang [39] have suggested that the increase in vector length to average environmental coordinate is an indicator of increased sensitivity of genotypes to environmental factors. The fact that the majority of genotypes are located far from the average environmental coordinate in some regions may also indicate that environmental factors are more challenging. For example, the Aegean Region and the Southern Marmara Region are accepted as a single region in variety registration trials since 2009 [26,40]. Therefore, these days the breeding programs focus on winter growth type cultivars in the Thrace Region, and alternative growth type cultivars in the Southern Marmara Region, and spring growth type cultivars in the Aegean Region. It is suggested that almost all of the variations in grain yield originate from environment and genotype × environment interaction since the Aegean & Southern South Marmara Region also have evolved agroecological differences within spring and alternative types. Other researchers also have similar observations for grain yield in their studies in several Anatolian locations [[40], [41], [42], [43], [44], [45]].

### 4.2 Quality characteristics

1000 kernel weight is an important parameter that is an indicator of flour yield [46]. Test weight plays a role in determining the market grades and prices of wheat [47]. 1000-kernel weight and test weight are characteristics that are under the influence of environmental factors as well as genotype [48,49]. The soil and climate of the cultivation area, crop technology, the prevalence of foliar diseases, pest attacks, agronomic practices, the chemical composition of the kernel, and the condition of the grains at harvest all have an impact on a genotypes test weight [50]. There are studies to predict flour yield with Artificial Neural Network modeling based on hectoliter weight, 1000 kernel weight, kernel size distribution, and grain hardness [51]. In terms of 1000 kernel weight, regional averages varied between 35.3 and 39.9 g, while a wider range (26.3–42.9 g) was determined on the basis of genotypes. The cultivars showed the highest standard deviation in terms of yield in the Thrace agroclimatic regional trials with the highest standard deviation in the average of 1000 kernel weight. The fact that the genotypes with the lowest and highest 1000 kernel weights were used in the trials in this region can be seen as the main factor affecting the yield. A wider range of variation was observed in the test weight based on the genotypes, as in the 1000 kernel weight. Especially the Southeastern Anatolia Region with the highest average values in terms of 1000 kernel weight, test weight, and protein contents.

Protein content is an important parameter in determining wheat quality and is affected by genotype and environment [52]. The regional averages of protein content varied by 1.3 %, while the difference in genotypes differed by 3.0 %. Protein content is controlled by genetic factors, but when the protein content of cultivars used in trials at more than one regions during comparison, e.g. cv. Ceyhan 99, performed differently in different regions. This showed phenotypic instability depending on the environmental conditions, which determined the genetic factors determining protein contents, but environmental factors had a big impact on gene expression among cultivars [53].

Zeleny sedimentation is an indicator of protein quality and is under the influence of genotype and environment. The share of genotypic effect is quite high [54]. The variation in the mean of genotypes in Zeleny sedimentation (30.6–66.6 mL) was quite high. Rheological properties of dough are important in determining protein quality and alveograph analyzes are used for this purpose [55]. Alveograph analyses are a reliable tool in determining flour quality and energy value, which has a special place in these analyses. Industrialists prevent this by blending to produce standard quality flour from products with different quality characteristics with the effect of genotype and environment [56]. The genotypes showed a wide range of values for alveograph energy value, between 120.8-433.0 × 10^−4^ J. Sanal et al. [57] reported that the alveograph energy value of red bread wheat in samples taken from different regions of Türkiye varied between 47.3-315.6 × 10^−4^ J.

### 4.3 High molecular weight glutenin subunits

Payne et al. [21] suggested that subunit 5 + 10 had the highest effect at *Glu-D1* in quality scores based on high molecular weight glutenin subunits. Later, subunits 1 and 2∗ at the *Glu-A1* locus, and then subunits 7 + 8 and 17 + 18 at the *Glu-B1* locus were reported effective. Galova et al. [58] suggested that 5 + 10 glutenin subunits are effective in high bread quality, followed by 1, 2∗ and 7 + 9 subunits. Karaduman et al. [59] has reported that subunit 7 had a weakening effect on gluten quality, while Subunit 17 + 18 was is similar to 7 + 8 but have a more pronounced effect on gluten strength. As seen in Table 11, subunit 7 had the lowest average in terms of protein contents, Zeleny sedimentation and alveograph energy values. The 17 + 18 subunit had the highest mean of alveograph energy value and the second highest mean in sedimentation after the 7 + 8 subunit.

Bedo et al. [60] reported that different genetic backgrounds can greatly influence the function of HMW glutenin subunits and consequently, the *Glu-Dl* locus in impacting flour quality, resulting in a complicated system regulating wheat breadmaking quality. High bread-making quality can be seen in genotypes with 2 + 12 HMW-GS as well as genotypes with 5 + 10 at *Glu-D1* subunits. It has been stated by many researchers that the 5 + 10 subunit is the most effective subunit on quality. It is seen in Table 11 that the 2 + 12 and 5 + 10 subunits at the *Glu-D1* locus had close average values in terms of the examined characters. This may be the reason why some genotypes had 2 + 12 subunits but showed high-quality values. For example, cv. Sagittario showed high-quality values despite having 1, 7 + 9, 2 + 12 subunits, and 7 quality scores. Akman et al. [61] reported that some wheat genotypes carried 2 + 12 subunit low quality at the *Glu-D1* locus, while the cv. Yellowstone carried 1 subunit at the *Glu-A1* and 5 + 10 subunits at the *Glu-D1*.

## 5 Conclusion

The study analyzed cultivars from various ecological regions in Türkiye, revealing that environment significantly impacts grain yield. The Central Anatolian Region, with its large cultivation area and rainfed conditions, had the lowest grain yield. Quality characteristics varied within regions, but regional averages remained consistent. The study suggests this multi-environment and multi-genotype study will serve as reference for future research.

## CRediT authorship contribution statement

**Bekir Aktaş:** Writing – review & editing, Writing – original draft, Validation, Supervision, Resources, Project administration, Methodology, Investigation, Formal analysis, Data curation, Conceptualization. **Halil İbrahim Gökdere:** Methodology, Investigation, Formal analysis.

## Data availability statement

Data will be made available on request. For requesting data, please write to the corresponding author.

## Funding

The authors acknowledge the support of Variety Registration and Seed Certification Center, Ankara and Department of Field Crops, Faculty of Agriculture, Yozgat Bozok University, Türkiye for carrying out the study.

## Declaration of competing interest

The authors declare that they have no known competing financial interests or personal relationships that could have appeared to influence the work reported in this paper.
